# Mr. Christie, on Puerperal Convulsion

**Published:** 1800-05

**Authors:** J. Christie

**Affiliations:** Deal


### *To the Editors of the Medical and Physical Journal.*

GENTLEMEN,

INCLOSED is an account of a Case of Puerperal Convulsion, with remarks on this affection, for insertion in the Medical Journal, should it appear worthy of your attention. I am,

GENTLEMEN,

With the greatest respect,

Your's, &c.

*Deal, April,* 10, 1800. J. CHRISTIE.

---

Having lately seen puerperal convulsion, under unhappy circumstances, terminate favourably, I have been induced to make out for the Medical Journal, the following account of this unfrequent, though most formidable symptom, as it appeared in the wife of a serjeant of the 27th regiment of foot.

M. Y. aged about 36, stout, well made, corpulent, of a ruddy, healthful complexion, is the mother of five children. All her labours, except the one which is the subject of the following description, were perfectly regular. The fourth child she had about three years previous to the last; during which interval she

ſhe had not any ſign of miſcarriage, although living conſtantly in the married ſtate.

In the beginning of laſt June, having for ſome time before felt herſelf impregnated, ſhe wiſhed, as was her cuſtom, to loſe ſome blood, and ten or twelve ounces were taken from her arm. During the period of utero-geſtation, ſhe enjoyed good health, although expoſed to conſiderable fatigue, wet and cold, during marches both in England and Holland. About the end of laſt November, ſhe was attacked with the ſymptoms of a ſlight cold, accompanied with a ſenſe of weight and oppreſſion about the præcordia. On the 1ſt or 2d of December, ſhe alſo complained of a heavineſs and pain of her eyes, with an intolerable giddineſs.

She then told her huſband, "ſhe was never this way before;" and thinking that her complaints were connected with her labour, which ſhe expected every day, ſhe reported herſelf to the medical perſon on duty at the regimental hoſpital. She was adviſed to be blooded; but not aſſenting readily, and this not being inſiſted upon, a doſe of ſalts was given to her, which, by the bye, ſhe never took. Between the 2d and 4th of December, ſhe ſhewed more than common ſigns of anxiety, repeating ſhe was never in this way before: ſhe complained, particularly, of a dizzineſs and defect of viſion, and thought particles were floating before her eyes. On the morning of the 4th, her former medical attendant was called to ſee her, who took ſome blood from her arm, and I believe ordered her feet to be bathed in warm water. Her huſband now began to think her conduct was becoming extremely ſilly and inconſiſtent: in the night of the 4th he was convinced of it; for then, without aſſigning any reaſon, ſhe ſuddenly ſtarted up and laid herſelf down, with her head towards the foot of the bed. Surpriſed at ſuch conduct, he got a light, and obſerved her to ſtare wildly, talking at the ſame time incoherently, and ſometimes moaning. About this time a tremor extended all over her body, and ſoon after ſhe became permanently ſenſeleſs. Early on the morning of the 5th of December ſhe had a general convulſive fit; another returned in little more than an hour with increaſed violence; and with an interval nearly of this period, the fits recurred, more or leſs ſevere, during the forenoon of the 5th. About two in the afternoon I was aſked to ſee her; I found the poor woman lying perfectly comatoſe, inſenſible to the ſtimuli of light, ſound, or drink; her breathing ſlow and laborious, pulſe ſmall and interrupted. I was informed, that the woman who acts as midwife in the regiment, had been deſired to give her a clyſter, and, I believe, to apply a bliſter ſomewhere; but ſo little ſuſpicion had there been, even even at this period, of the ſymptoms being connected with labour, that there was not even an attendant ordered, nor was any examination ſuggeſted or attempted. Should this ſtatement be ſuſpected, for a moment, to ariſe from any other motive than that of *humanity*, let thoſe concerned take a poſture of defence; let the advance be upright; honeſtly ſhall I combat; impartiality, truth, and the world being umpires.

On examination, I found the os tincæ dilated, and abundant relaxation of the contiguous parts; the head of the child could be felt. I had been but a few minutes in the room, when the attendants told me, from the wreathing of her body and ſigns of uneaſineſs, that ſhe would ſoon have another fit: ſoon after, a fit of general convulſion, with diſtorted and blackened countenance, foaming of the mouth, laſting I ſuppoſe for more than a minute, ſupervened; the ſame obſtinate ſtupor continuing after the fit was over. I ordered the nurſe merely to watch the advancing labour, while I took away twelve or fourteen ounces of blood from her arm; from this, I could perceive no other apparent change than a leſſening of the redneſs in the countenance. The fits, for ſome time previous, had come on every half hour, and ſometimes even in leſs time; it was evident they were connected with the action of the uterus. The delivery of a ſtout, full grown, healthy looking child, was accompliſhed with aſtoniſhing rapidity, and without artificial aid, within an hour of my firſt ſeeing her; a ſecond pain expelled the placenta, which was large and natural, and there was no alarming hæmorrhage. A moſt profuſe ſweat followed the fit in which the child was born; and now there appeared to be ſome remiſſion in the general tumult of the ſyſtem: ſhe remained quiet, but perfectly comatoſe. From the occaſional wreathing of her body, and preſſing downwards of her hands, I began to ſuſpect ſomething was wrong, or ſomething more was coming. An hour after delivery ſhe had another fit, equal in ſeverity to any of the former; I repeated the blood-letting, and now took the blood from the temporal artery, which flowed abundantly. On a ſecond examination, I found the os tincæ very much dilated, and I felt ſomewhat that made me believe there was either a polypous tumour, a falſe conception, or a partial invertion of the uterus; it felt like the head of a child ſurrounded with ſome gelatinous matter or clotted blood. The ſwelling and motion of the abdomen too, were not of that equable kind which follows a common labour. I was extremely anxious: I was not even certain but there was a ſecond child; and being not much converſant in this branch of practice, I deſired another gentleman to be ſent for; and Mr. Mackeſon, ſurgeon in Deal, came in about about three hours after the delivery; when she had another fit of convulsion, though not equal in severity to any of the former ones. The tumour we felt still pressing upon the cervix uteri, but it had become less distinct, and the roundness of the abdomen assumed a more regular and diminished appearance; he satisfied me there was no second child. A clyster had been given shortly before this, and immediately after the last fit, which appeared to excite several pains, but those were either of the nature of after-pains, or in consequence, probably, of some convulsive action in the uterus itself; which I think, in this case, had suffered a *partial inversion*, forming the tumour mentioned.

As her pulse now rose both in frequency and fullness, as her countenance still appeared much flushed, and as she still continued to lie in an apoplectic state, we agreed to take away more blood, should the fits return. About ten o'clock in the evening, she lay in the same profound stupor, but without any return of the convulsion. Saw her on the morning of the 6th, about nine o'clock; she had one fit in the course of the night, and it was in less violence than any of the preceding. Stupor remains—breathing hurried—pulse about 110—skin hot and moist—face flushed. Ten ounces more of blood were taken from the arm; she retracted her arm as in pain, on the introduction of the lancet. At 8, P. M. oppression in breathing lessened surprisingly, as well as the frequency of her pulse and redness of her countenance—stupor continues—her head is shaved, and a blister applied.

7th December, mid-day. Blister has been dressed—has had no return of the fit—stupor seems to wear off—she looks up occasionally on being spoken to, but immediately afterwards shuts her eyes, as if desirous of remaining undisturbed—pulse upwards of an hundred, and feeble—skin moist and less hot than yesterday. The lochia, of a natural appearance, have been in less than ordinary quantity; is now ordered to take a tonic mixture, in which there is aromatic confection and vitriolic æther, along with a gill of mulled wine, three times a day.

Nine o'clock in the evening. Skin still agreeably moist with little heat—pulse still upwards of an hundred in the minute. On rousing her, she immediately seems desirous, as before, of remaining undisturbed; is observed to sigh frequently and deeply, as if from a sudden surprise; she also is seen often to stretch forth and draw up her limbs, as if she felt ease from this sort of motion. Her pulse seems to have a curious peculiarity in it: for thirty or forty strokes, sometimes for half a minute, it beats small and weak; and afterwards, for a similar time, it rises fuller and stronger, resembling as it were, the ebbing ing and flowing of the waves of the ocean. In other caſes, where the powers of the conſtitution had been previouſly much exhauſted and deranged, and where they appeared to be gradually recruiting, I think I have more than once obſerved this waving ſort of feel in the pulſe; ſometimes, indeed, unequal preſſure of the finger may give one this ſenſation; but in this caſe, having felt both arms with attention, it was certainly diſtinct, and that without any evident intermiſſion.

Morning of the 8th of December. Stupor continues wearing off—pulſe leſs frequent, ſtill feeble—ſkin moiſt—bowels open; on deſiring her to ſhew her tongue, ſhe attempted it by opening her mouth. Her countenance now ſhews intelligence combined with anxiety; when counting the pulſations at her wriſt, ſhe was obſerved to look ſtedfaſtly, firſt at the watch, then at me, and then at the by-ſtanders, communicating a ſenſation peculiarly agreeable, and eaſier to be felt than deſcribed. The people around her now obſerve, that ſhe ſeems anxious to ſay ſomething, but has not yet uttered a word more than the monſyllables yes and no. Injunctions given to keep her very quiet; and eſpecially, when ſhe aſks any queſtion reſpecting her ſituation, to inform her of it in as cautious, gradual a manner as poſſible, to guard the mind againſt any ſudden exertion or ſhock. Ordered to continue the tonic remedies.

Morning of the 9th of December; pulſe ſtill feeble and frequent, ſkin moiſt, heat natural, tongue clean; now underſtands perfectly what is ſaid to her, to which ſhe gives diſtinct anſwers, but ſeemingly with reluctance; can now ſwallow drink readily, and can even take a little light food. From this period ſhe gradually recovered both the functions of the mind and the ſtrength of the body. The ſecretion of the milk was for ſome days nearly gone. She complains of pains through her body, particularly in her limbs, and compares them to thoſe pains felt after any long continued violent exerciſe.

13th of December; found her continuing in a ſtate of convaleſcence, but very weakly; and complaining ſtill of the pains in her limbs; has yet no recollection of any thing that happened during her illneſs.

22d of December; ſhe continues gaining ſtrength; is not yet entirely free from the pains of the limbs: ſhe came to recollect being reported to the medical perſon who firſt ſaw her, but beyond this period knows nothing of what happened in her illneſs. The ſecretion of the milk returned abundantly with her ſtrength. She is now, at the time I am writing, in perfect health and ſpirits, and very thankful; for ſhe roſe again, although three days of her life have been buried in the darkneſs of death.

I hope the hiſtory of the foregoing caſe will not be without its uſe; it is indeed a matter of regret that we know nothing at all of the ſtate of the os internum at the time of the approach of the fits; and the propriety of aſſiſting *promiſcuouſly*, by direct means, the wiſhed-for dilatation, is a ſubject well known to have the moſt able advocates; while others, of no leſs reſpectability, have doubted the propriety of the practice here ſpoken of.* It is quite foreign to my intentions, and indeed altogether beyond my preſent ſphere, to attempt any deciſion on the queſtion, What caſes of labour, accompanied with convulſion, require direct artificial aſſiſtance; eſpecially after the ample and ſatisfactory directions given in books on Midwifery: all I now aim at, is to inculcate the propriety of the evacuating plan, and particularly blood-letting, as I think I have, in more caſes than one, prevented puerperal convulſion ſupervening, by the timely, free uſe of the lancet, where the approach of this dreadful ſymptom was not indiſtinctly marked, and in habits too where the propriety of blood-letting might have been much queſtioned. Moſt writers recommend an early attention to bleeding and purging, as the means moſt likely to prevent puerperal convulſion. The unfrequency of the ſymptom has probably prevented their recommendation from being ſufficiently attended to, and I have reaſon to ſuſpect it has been too often entirely overlooked. In the caſe before us, I was ſo much convinced of the utility of blood-letting, even after the fits had commenced, and after the delivery was accompliſhed, that I have no heſitation in believing, the fits would either have been altogether prevented, or at leaſt highly modified, had the lancet been largely uſed when the woman firſt reported herſelf; as I conſider that was the period the conſtitution received the warning of the impending danger; that the ſymptoms of oppreſſion, the difficulty of breathing, the pain of the eyes and head were the true fore-runners of the convulſions; that this was the moſt proper period for bleeding, and which ſhould therefore put us on our guard in ſimilar caſes. With regard to the appearance of the blood in the preſent inſtance, if that is to be conſidered as an infallible guide, it was even in our favour, there being on the blood a ſlight buffy coat, except the blood drawn from the artery, which ſhewed a florid ſmooth maſs, more homogeneous in its appearance than the venal blood. In pregnancy it has been generally obſerved, that the blood ſhews a morbid appearance; hence it has been ſuppoſed, a proceſs

* Among the former may be reckoned Dr. John Clarke, and with the latter I would claſs Dr. Thomas Denman.

proceſs in ſome reſpects ſimilar to inflammatory action takes place. From the appearances of blood drawn in inflammatory affections, I have been led to imagine, that there is leſs diſpoſition in arterial than in venal blood to ſhew a buffy coat, and that when the buffy coat does appear on the ſurface of the arterial craſſamentum, it aſſumes a leaden coloured ſhade, or the hue is darker than the venal buff. Will this appearance be produced by the lighter colour of the arterial craſſamentum? or does the buffy coat of venal blood prevent the air acting upon the ſurface of its darker craſſamentum? In this place I beg to obſerve, that within the laſt twelve months, and eſpecially ſince laſt autumn, there has been an epidemic fever prevailing among the troops, and which has carried off inconceivable numbers. I underſtand, a diſeaſe of this kind has alſo been frightfully frequent in civil life; I have reaſons for ſuſpecting that the nature of this diſeaſe, (which I call "Typhus Pneumonoides") has not unfrequently been miſtaken. Sometimes, from the inſidious nature of the ſymptoms, it has been called, and treated, as a ſimple nervous fever, while probably the pulmonary affection killed; and again, that it has been ſuppoſed to be ſimple pneumonia, while a ſpecific contagion of ſtrongly debilitating powers had been the ſource of the diſeaſe and the cauſe of the modification of its ſymptoms. At preſent, I am employed in arranging for the preſs, materials for a little work on the nature and cure of this diſeaſe, together with appearances on diſſection. In this, I hope I ſhall be able to ſhew the neceſſity of copious blood-letting, together with tonics and ſtimulants, and ſometimes even to be employed at one and the ſame period, although the early uſe of the former will in moſt inſtances ſuperſede that of the latter. No means, perhaps, have been ſo frequently and ſo variouſly treated of and diſputed upon as blood-letting; but I believe all experienced practitioners, and men above "ſerving any theory" but that reſulting from attentive obſervation, have agreed on its propriety, nay neceſſity, in certain diſeaſes; — one of your correſpondents, indeed, whoſe reaſoning (fortunately perhaps for the community) will make but few converts, and which has, in my opinion, very juſtly drawn a reply from another gentleman, ſeems to fancy that bleeding may be entirely expelled from practice, for "he has no heſitation in aſſerting, that it is not neceſſary in any complaint with which we are acquainted." Another of your correſpondents, in the ſame month's Journal, with an expediency equally his own, in curing the *bloody flux* by flannel rollers and ſticking plaſters, has frequently taken away forty, fifty, and ſixty ounces of blood in a couple of hours, by which many valuable lives have been ſaved, and in a *diſeaſe too where all kinds of me-*

*dicines do miſchief!* What a difference of opinion may exiſt between two *Licentiates in Phyſic*, on a *ſubject* apparently not quite *incomprehenſible. The thing ſpeaks for itſelf.* It is much to be lamented, that writers on medical matters are too apt to be intoxicated with thoſe notions which have the *air* of originality. Sly vanity, or frantic ambition, comes ſtalking abroad in the dark under the veil of candour, becoming blind to every ray of light, to every ſhade of beauty, but thoſe that fall on the little conceited ſcenes. When thoſe conceits are pent up in a corner, out of the way of harm, it may be rather cruel to pull them down; but when placed in a conſpicuous ſituation, to dazzle like new tinſel at a diſtance, to diſappoint, perhaps fatally to deceive; it then becomes high time the materials ſhould be analyzed and compared with the ſolid ſtructure of worth, of old induſtry, of extenſive experience, of candid reaſoning, of ripened talents: and in the cure of diſeaſes, perhaps thoſe who affect *moſt* to overlook the powers inherent in Nature, ſtand *moſt* in want of her aſſiſtance!

