# Errata

**Published:** 1800-05

**Authors:** 


					p. 450, 1. 3, for ymt, read May.

				

